# Supplementary material for: Microbiome-Derived Metabolites Shape CD4+ T-Cell Differentiation and Immune Aging in Chronic HIV-1 Infection
Source: bioRxiv. 2026 Jan 14:2026.01.13.699280. Preprint. [Version 1] doi: 10.64898/2026.01.13.699280 (PMC12871317; doi:10.64898/2026.01.13.699280)

bioRxiv preprint doi: https://doi.org/10.64898/2026.01.13.699280; this version posted January 14, 2026. The copyright holder for this preprint (which was not certified by peer review) is the author/funder, who has granted bioRxiv a license to display the preprint in perpetuity. It is made available under aCC-BY 4.0 International license.

Supplementary Figure 1. PCS concentrations in plasma and CD4$^+$ T-cells from PLWH reveal tissue-specific accumulation and lack of correlation.

(**A**) Mass spectrometry was used to quantify PCS concentrations in paired plasma and CD4$^+$ T-cell samples from 50 PLWH. The mean PCS concentration in plasma was 11.82 µM, whereas the mean cell-associated concentration in CD4$^+$ T-cells was 0.025 µM. **(B)** Spearman correlation analysis between plasma and CD4$^+$ T-cell PCS concentrations. **(C)** Cell-associated PCS accumulation in CD4$^+$ T-cells is shown from a separate experiment in which cells were incubated in vitro with 50 or 100 µM PCS for 1, 3, or 6 days, with or without TCR stimulation.

Supplemental Figure 2. Marker distribution across CD4$^+$ T-cell clusters used to define populations in Figure 2C.

t-SNE projection of CD4$^+$ T-cells from PLWH showing single-marker expression intensities across 30 FlowSOM-defined clusters. Each plot represents the expression of the indicated surface or intracellular marker (CD45RA, TCF7, FOXP3, PD-1, CD25, CD71, Ki-67, CD127, CCR7) across the CD4$^+$ T-cell landscape. Expression intensities are color-coded from low (blue) to high (red). These marker distributions were used to assign phenotypic identity to CD4$^+$ T-cell subsets shown in **Figure 2C**.

Supplementary Figure 3. Transcriptomic response to PCS in proliferating and non-proliferating CD4$^+$ T-cells.

Heatmaps show hierarchical clustering of gene expression profiles (Z-score normalized) across PCS doses for proliferating (left) and non-proliferating (right) CD4$^+$ T-cells. Transcriptional changes across both subsets showed broadly similar PCS-induced expression patterns. Based on this similarity, downstream transcriptomic and proteomic analyses were focused on the proliferating (CTV^{low}) CD4$^+$ T-cell subset.

bioRxiv preprint doi: https://doi.org/10.64898/2026.01.13.699280; this version posted January 14, 2026. The copyright holder for this preprint (which was not certified by peer review) is the author/funder, who has granted bioRxiv a license to display the preprint in perpetuity. It is made available under aCC-BY 4.0 International license.

Supplementary Figure 4. PCS modulates cytokine production and impairs Th1/Th2 polarization in CD4$^+$ T-cells.

(A) Heatmap of 40 cytokines measured in cell culture supernatants by multiplex detection system (MDS) from 5 healthy donors (HC1–HC5). PBMCs were stimulated with anti-CD3/CD28 and cultured in the presence of increasing concentrations of PCS (0, 10, 50, or 100 μM) for 12 hours, 24 hours, or 6 days. Cytokines were hierarchically clustered based on expression patterns. **(B)** Cluster analysis of cytokine profiles identified distinct PCS-responsive modules. Cluster 1 (IL-7, SDF-1a, TGF-β3) was upregulated at 24 hours with 100 μM PCS. In contrast, clusters 2, 4, 6, and 7, which include proinflammatory and T helper–associated cytokines (e.g., IL-6, TNF-α, IL-17A, IL-4, IL-5, IL-13), were significantly downregulated by PCS at multiple timepoints. Statistical significance determined by paired t tests. **(C)** PBMCs were stimulated with anti-CD3/CD28 for 6 days in the absence or presence of 100 μM PCS under non-polarizing (top left two panels), Th2-polarizing (top right two panels), or Th1-polarizing (bottom four panels) conditions. Cells were gated on CD4$^+$ T-cells and analyzed for the expression of transcription factors GATA3 (top panels) and T-bet (bottom panels). The percentage of positive cells and the corresponding mean fluorescence intensity (MFI) values are shown on each plot. Data in **(C)** are representative of three independent experiments.

Supplementary Figure 1

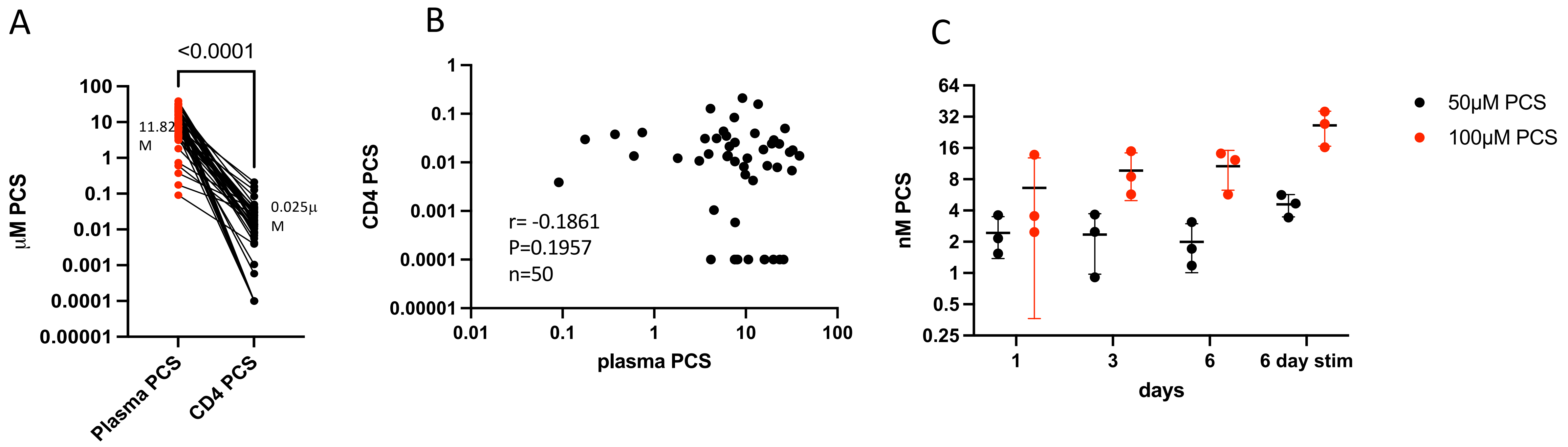

Supplemental Figure 2

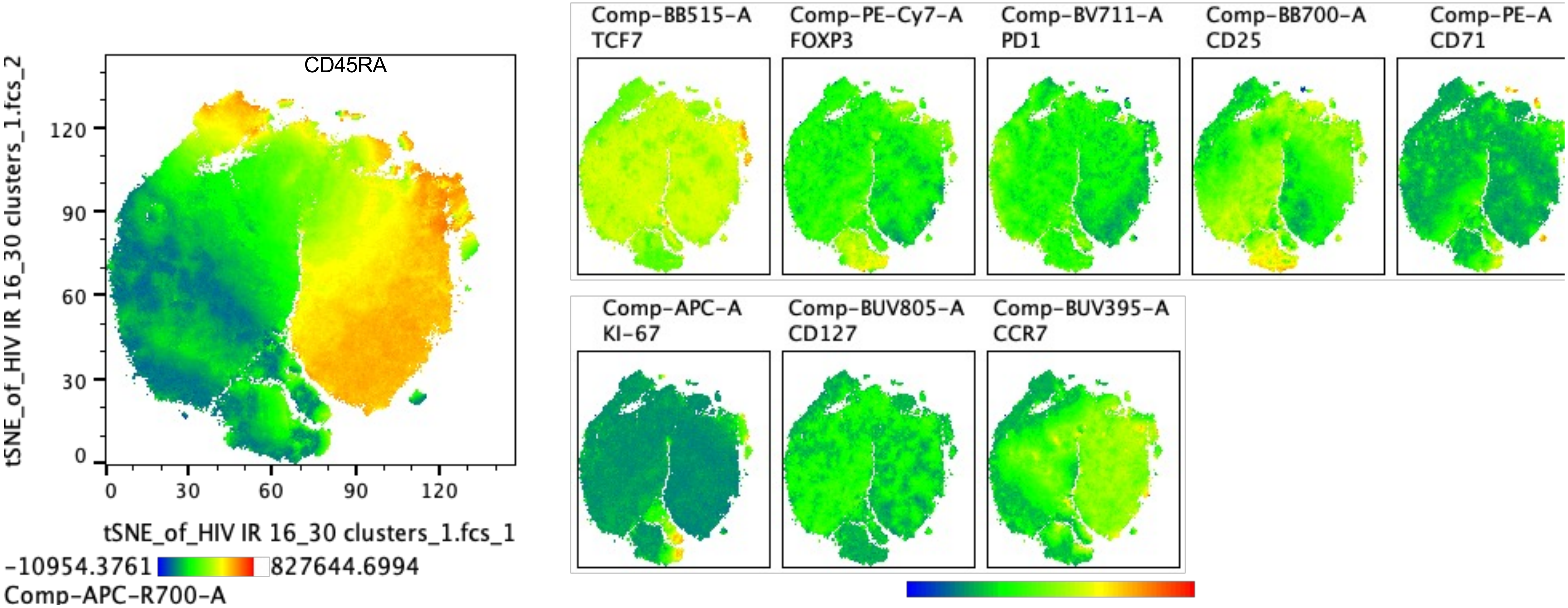

Supplemental Figure 3

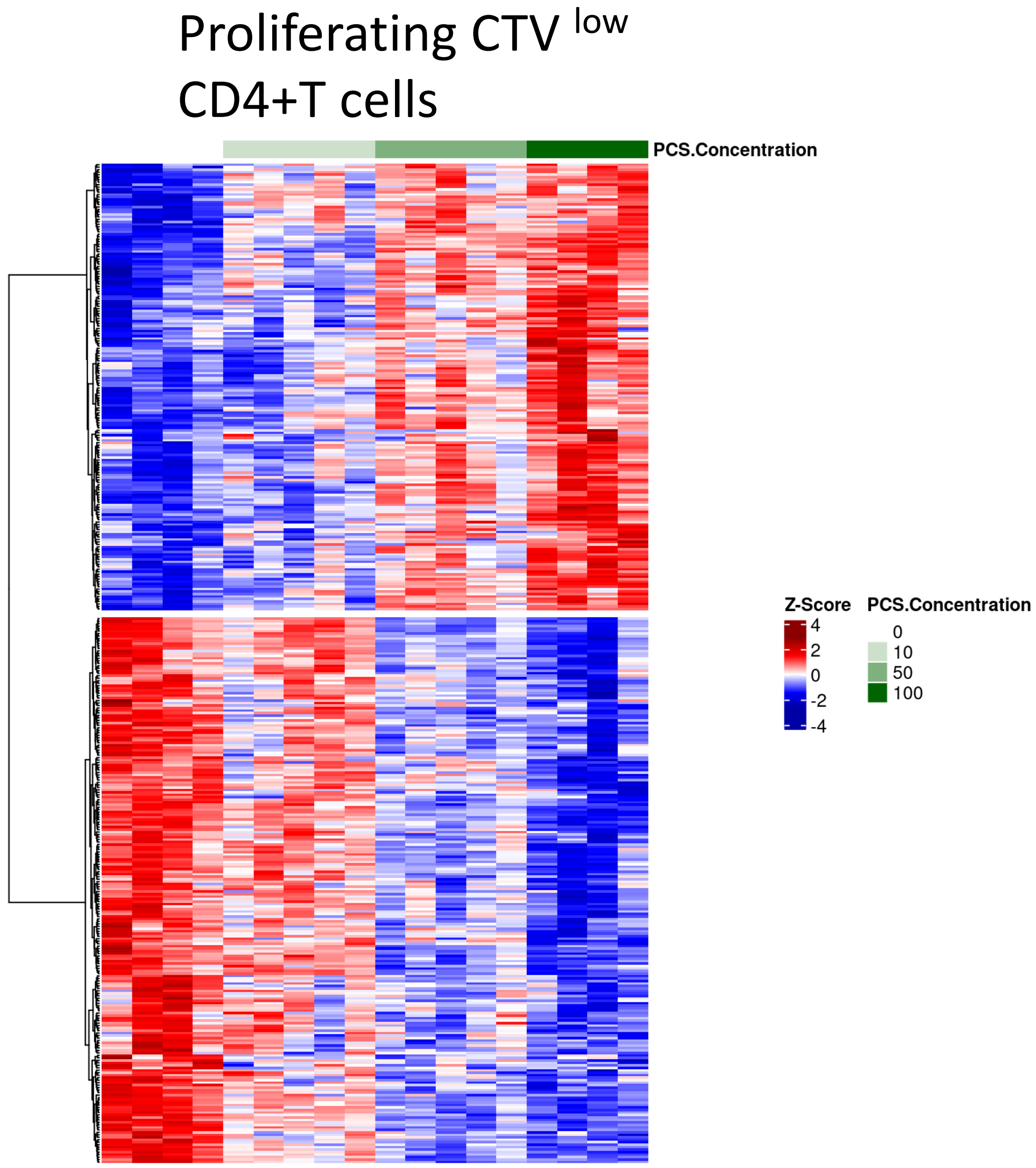

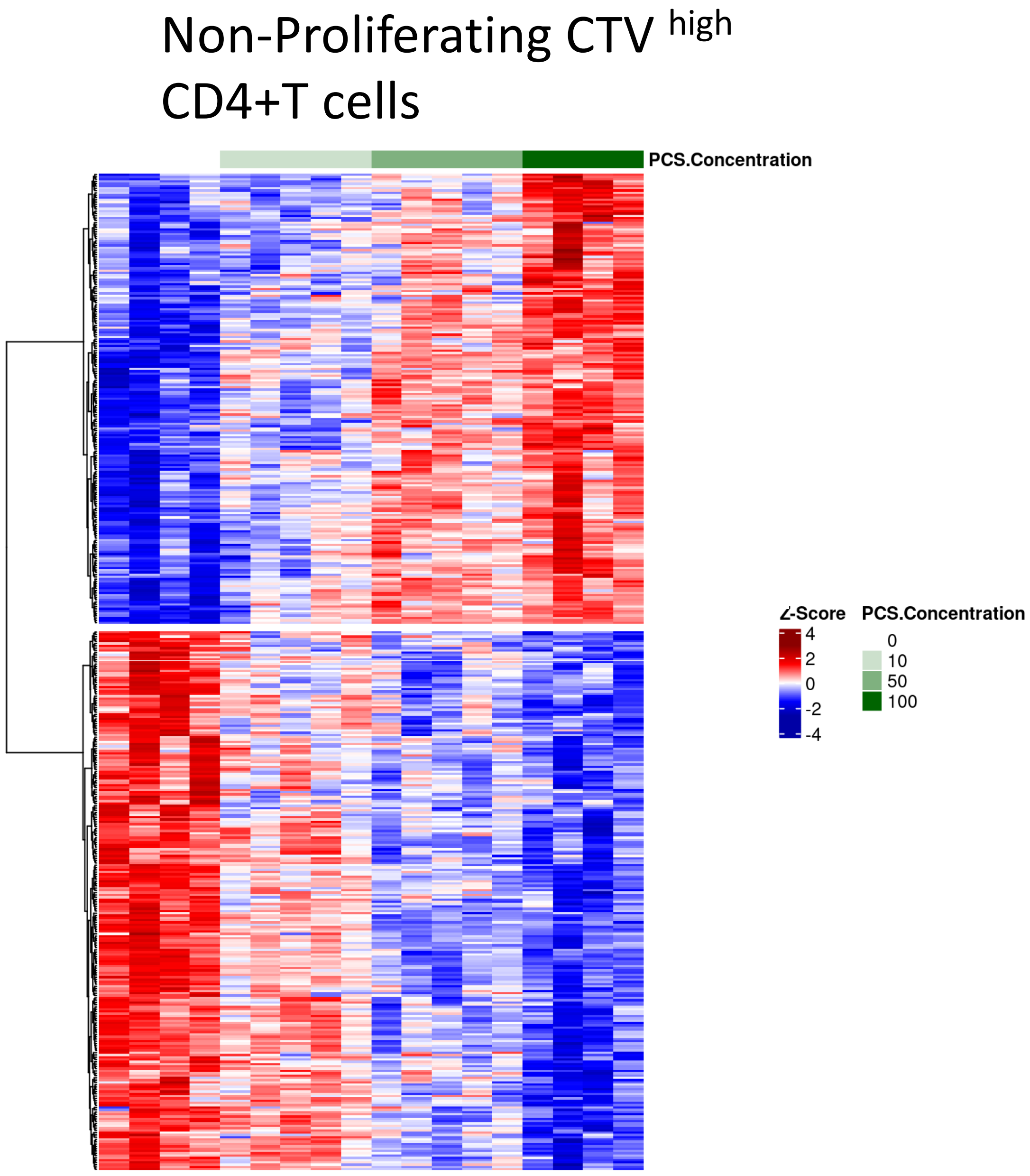

Supplementary Figure 4

A

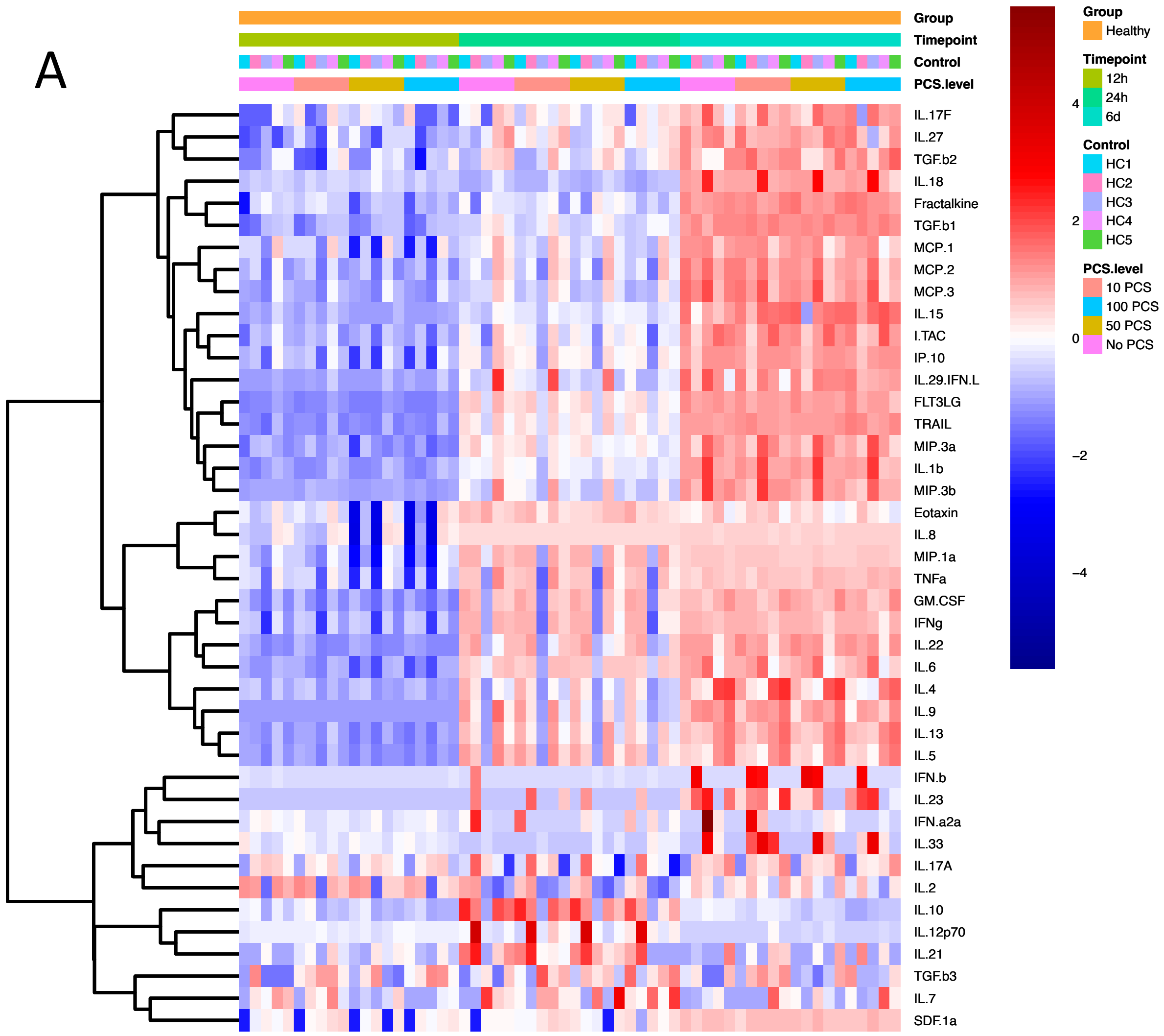

B

	Timepoint	p-value	
**Cluster 1: IL-7, SDF-1a, TGF-B3**	24 hr	0.042	Up in 100
**Cluster 2: Eotaxin, iL-6, iL-8, MCP-1, MIP-1a, TNFa**	24 hr	0.09	Down in 100
**Cluster 4: FLT3LG, GM-CSF, I-TAC, IFNg, IL-13, IL-22, IL-4, IL-5, IL-9, IP-10, TRAIL**	12 hr	0.025	Down in 100
**Cluster 4: FLT3LG, GM-CSF, I-TAC, IFNg, IL-13, IL-22, IL-4, IL-5, IL-9, IP-10, TRAIL**	24 hr	0.069	Down in 100
**Cluster 4: FLT3LG, GM-CSF, I-TAC, IFNg, IL-13, IL-22, IL-4, IL-5, IL-9, IP-10, TRAIL**	6 d	0.061	Down in 100
**Cluster 6: IL-10, IL-12p70, IL-21**	24 hr	0.087	Down in 100
**Cluster 7: IL-17A, IL-2**	12 hr	0.023	Down in 100
**Cluster 7: IL-17A, IL-2**	24 hr	0.066	Down in 100
**Cluster 7: IL-17A, IL-2**	6 d	0.024	Down in 100

C

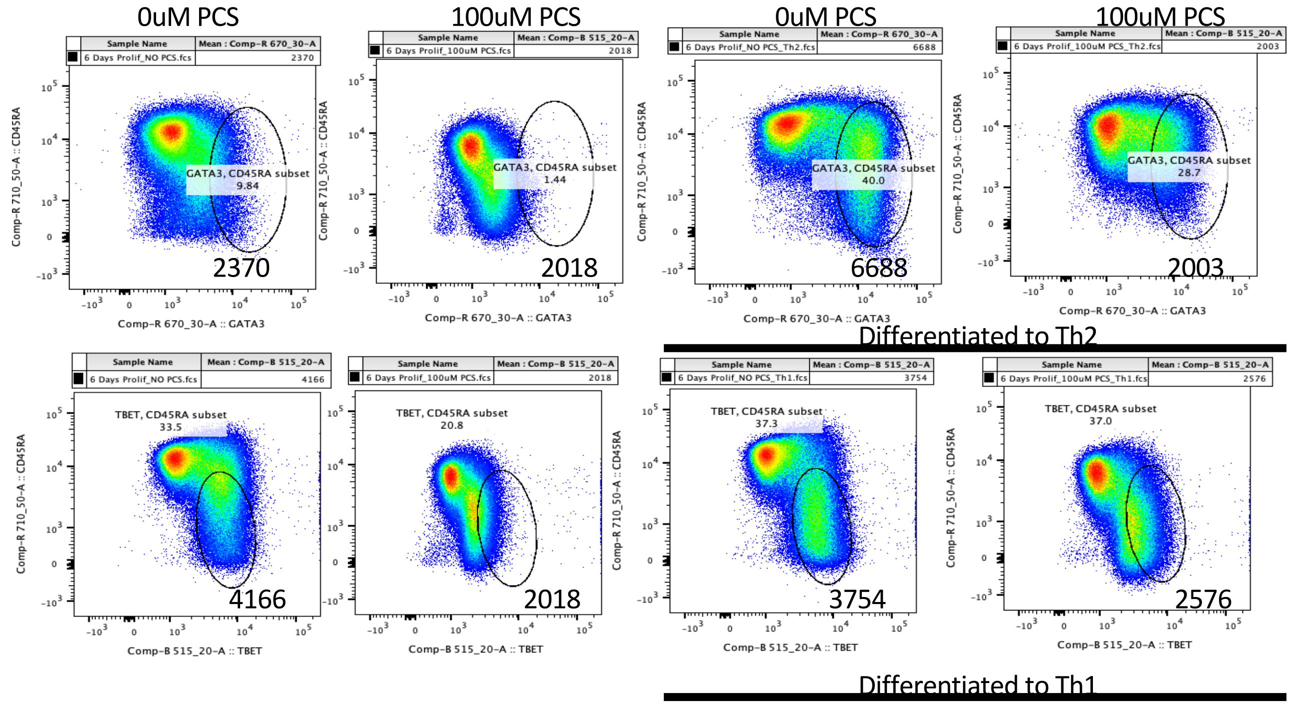

Supplement: Supplement 1 [file NIHPP2026.01.13.699280v1-supplement-1.pdf]
